# Functional Implications of Epstein-Barr Virus Lytic Genes in Carcinogenesis

**DOI:** 10.3390/cancers14235780

**Published:** 2022-11-24

**Authors:** Lee Fah Yap, Anna Kang Chee Wong, Ian C. Paterson, Lawrence S. Young

**Affiliations:** 1 Department of Oral & Craniofacial Sciences, Faculty of Dentistry, University of Malaya, Kuala Lumpur 50603, Malaysia; 2 Oral Cancer Research and Coordinating Centre, University of Malaya, Kuala Lumpur 50603, Malaysia; 3 Warwick Medical School, University of Warwick, Coventry CV4 7AL, UK

**Keywords:** EBV, lytic genes, oncogenesis, gene expression

## Abstract

**Simple Summary:**

Epstein-Barr virus (EBV) was the first human tumor virus to be discovered and is a causative agent for several cancer types of epithelial and lymphoid origin. EBV has two life cycles comprised of latent and lytic phases. The lytic cycle is when new virions are produced, whereas the latent cycle is a state of persistent infection without productive viral replication. It has been recognized that latent infection is the predominant mode of infection in EBV-associated cancers and the expression of a restricted set of latent genes drives disease development. However, we now know that several lytic genes are also expressed in EBV tumors, suggesting a critical role for these genes in tumorigenesis. Here, we summarize the current evidence as to how EBV lytic genes might contribute to EBV-driven oncogenesis.

**Abstract:**

Epstein-Barr virus (EBV) is associated with a diverse range of tumors of both lymphoid and epithelial origin. Similar to other herpesviruses, EBV displays a bipartite life cycle consisting of latent and lytic phases. Current dogma indicates that the latent genes are key drivers in the pathogenesis of EBV-associated cancers, while the lytic genes are primarily responsible for viral transmission. In recent years, evidence has emerged to show that the EBV lytic phase also plays an important role in EBV tumorigenesis, and the expression of EBV lytic genes is frequently detected in tumor tissues and cell lines. The advent of next generation sequencing has allowed the comprehensive profiling of EBV gene expression, and this has revealed the consistent expression of several lytic genes across various types of EBV-associated cancers. In this review, we provide an overview of the functional implications of EBV lytic gene expression to the oncogenic process and discuss possible avenues for future investigations.

## 1. Introduction

EBV is the most prevalent virus infection in humans with approximately 95% of the world’s population sustaining an asymptomatic life-long persistent infection. The discovery that EBV is the causative agent of infectious mononucleosis (IM) represented a breakthrough in our understanding of primary EBV infection in healthy individuals [1]. A wealth of data demonstrates that EBV is transmitted through saliva with subsequent infection of naïve B cells in the naso/oropharynx. EBV induces proliferation of these B cells into activated B blasts that escape primary T-cell responses and undergo a germinal centre reaction driven through a series of viral latency programs. This culminates in EBV residing in the resting memory B cell pool, thereby establishing a persistent latent infection [2,3]. The EBV lytic cycle is switched on when EBV-infected memory B cells differentiate into plasma cells or when the virus enters differentiating epithelial cells, and this results in the production of new virions for transmission to other hosts [4].

Disruption of this intimate host-viral interaction can lead to malignant transformation. Unlike the pathogenic process in EBV-induced lymphomagenesis where the virus appears to be the initiator, EBV contributes to the development of epithelial tumors, namely nasopharyngeal carcinoma (NPC) and EBV-associated gastric carcinoma (EBVaGC), as a consequence of the aberrant establishment of virus latency in epithelial cells with existing pre-malignant changes that disable the normal differentiation process [5].

The EBV genome comprises approximately 172 kb and encodes around 100 open reading frames that are generally divided into two categories, latent and lytic genes. A limited subset of viral gene products, the so-called latent proteins, consists of six nuclear antigens (EBNAs 1, 2, 3A, 3B, 3C, and -LP) and three latent membrane proteins (LMPs 1, 2A, and 2B). Over the years, these latent proteins have been regarded as key molecules in the pathogenesis of EBV-associated cancers by regulating various critical cellular processes and pathways [5]. The contribution of these latent proteins to the oncogenic process remains the subject of intense study. By contrast, although there are more than 80 EBV lytic genes, for the majority of these genes far less attention has been paid to their possible roles in oncogenesis. Nonetheless, it is now becoming increasingly clear that the EBV lytic phase also plays an important role in EBV-driven carcinogenesis. In particular, an early lytic gene, BARF1, is now recognized as being consistently expressed in NPC and EBVaGC, highlighting the need to be more open-minded about the possible contribution of EBV genes outside of those traditionally associated with latent infection. This also stresses the need to obtain more comprehensive EBV gene expression profiles in EBV-associated cancers. The advent of next generation sequencing technology has allowed the discovery and interrogation of exogenous pathogens associated with various types of cancers. RNA sequencing (RNAseq) captures genetic and transcriptional profiles of cancer cells, which can be leveraged to examine not only the host genome but also pathogen genomes infecting host cells. In recent years, an increasing number of research groups have used RNAseq analysis to determine the transcriptomic profiles of EBV-associated cancers [6,7,8,9,10,11,12,13,14,15,16,17,18,19]. Although the pattern of EBV gene expression varies between malignancies, these studies consistently demonstrated that a number of EBV lytic genes are more widely expressed than previously recognized, and the expression levels of some of these genes are similar to those of latent genes. Here, we provide an overview of known mechanisms by which EBV lytic genes could contribute to the oncogenic process. We focus our attention on early and late lytic genes whose expression has consistently been detected in biopsies from different EBV-associated tumors using RNAseq analyses.

## 2. EBV Lytic Cycle

**Figure 1:**
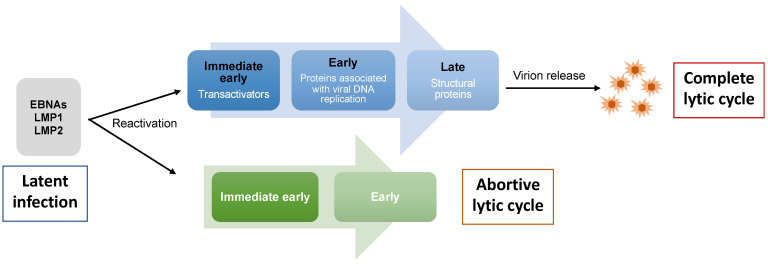
EBV lytic cycle. During latent infection, a restricted number of EBV latent genes are expressed in infected cells. Upon reactivation, cells that undergo complete lytic cycle express the immediate early genes (*BZLF1*, *BRLF1*), followed by early genes required for viral genome replication. Late genes that encode mainly viral structural proteins are then expressed and this is followed by the production of new viral particles. However, some cells that are reactivated undergo abortive lytic cycle where they express *BZLF1* together with one or more early genes in the absence of late genes, and thus do not produce mature virions.

As with many viruses that can establish a persistent infection, the switch from a latent to lytic EBV infection leads to virus replication with the production of new viral particles, and eventually the lysis of productively infected cells. The EBV lytic cycle can be activated in virus-infected B cells and epithelial cells by diverse stimuli in vitro, including phorbol ester and histone deacetylase inhibitors (HDACs) [20,21]. While the precise in vivo conditions responsible for this process remain to be explored, it is intimately related to the differentiation of both B cells and epithelial cells. The lytic cycle is characterized by the expression of a large number of viral proteins and is divided into three temporal and functional stages: immediate early, early, and late [22]. Zta (encoded by *BZLF1*) and Rta (encoded by *BRLF1*) are the immediate early transcription factors in charge of activating the cascade of EBV lytic gene expression. Early genes mostly encode proteins associated with virus DNA replication, and late gene products mainly support the formation of viral particles (Figure 1).

Zta and Rta synergistically stimulate the expression of multiple early lytic genes, including those that encode the components of the core replication machinery: BALF5 (the DNA polymerase), BALF2 (the single-stranded DNA-binding protein homolog), BMRF1 (the DNA polymerase processivity factor), BSLF1 (the primase homolog), BBLF4 (the helicase homolog) and BBLF2/3 (a potential homolog of the third component of the helicase-primase complex) [23]. In addition to its function as a transactivator, Zta binds to oriLyt (lytic origin of DNA replication) to initiate EBV DNA replication [24].

Traditionally, it has been thought that the latent and lytic cycles are two mutually exclusive mechanisms that contribute to lifelong infection by EBV. The expression of a restricted set of latent genes is compatible with persistent infection because it allows the virus to escape from host immunity. By contrast, the expression of lytic genes during viral replication is primarily found in the epithelium of oropharynx and salivary glands and it is thought to promote the spread of new viral particles [25,26]. It also likely that full lytic infection can occur periodically in B cells, but this has not been demonstrated in vivo. There is a significant immune response, both humoral and cell-mediated, to EBV lytic antigens in all individuals who are infected with EBV. This demonstrates that lytic infection is a continuous feature of virus infection, providing a constant stimulus to the immune system.

A possible tumorigenic role for lytic genes has emerged since the concept of “abortive lytic cycle” was proposed [27]. Zta preferentially binds and transactivates methylated promoters [28]. While a complete lytic cycle (i.e., production of progeny virus) immediately post-infection cannot be achieved while the viral genome is not methylated, Zta is still able to induce the expression of several early genes. The expression of *BZLF1* together with one or more early genes in the absence of lytic genes primarily encoding late structural proteins is termed the abortive lytic cycle (Figure 1).

## 3. Expression of EBV Lytic Genes in Tumors

The co-existence of latency and abortive lytic cycles has been documented in EBV-associated malignancies, including NPC, EBVaGC, and BL [29,30,31]. These studies consistently reported the presence of *BZLF1*/*BRLF1* and early genes, while late genes were either detected at low frequency or low levels. Unexpectedly, despite evidence for an incomplete lytic cycle, the expression of some late lytic genes has been detected in tumor samples by RNAseq analyses [6,7,8,9,10,11,12,13,14,15,16,17,18,19]. Some late genes have been classified as “leaky” because they are expressed at low levels early in the lytic phase and further increased following EBV DNA replication [32]. However, this does not explain the presence of some “true late genes” in tumor samples whose expression is usually dependent upon lytic DNA replication. Whether EBV acquires a yet-to-be-identified strategy to express late genes that could contribute to the development of disease remains to be determined.

Although there have been reports demonstrating the detection of lytic proteins in a variety of EBV tumors, comprehensive characterization of EBV transcriptomic profiles has only been made possible with the advent of RNAseq analysis. Due to technical and/or clinical challenges, most previous studies have used bulk tumor tissues. Thus, it remains to be determined whether such virus lytic RNA expression was derived from EBV-positive tumor cells or surrounding EBV-positive B cells that happen to be undergoing lytic cycle. Nonetheless, given that several independent research groups reported similar observations across different cancer types, it is now clear that EBV-associated tumors often express a wide-range of lytic genes that could play pivotal roles in EBV oncogenesis. This notion is reinforced by single-cell transcriptomic analysis showing co-existence of EBV latent and lytic genes in tumor cells isolated from primary NPC tissues [17]. The EBV lytic genes that have consistently been detected in biopsies from different tumor types using RNAseq are listed in Table 1. Of these, a number have also been detected at the protein level in tissues or cell lines, or antibodies against them detected in patient specimens (Table 2). As the tumorigenic roles of the two immediate early genes *BZLF1* and *BRLF1* have been discussed and reviewed previously [33,34], we focused our attention on early and late genes with known mechanisms that are relevant to EBV oncogenesis.

## 4. Functional Implication of EBV Lytic Genes in Tumorigenesis

### 4.1. Immunomodulation and Immune Evasion

EBV has developed mechanisms to regulate and evade immune detection, and this is believed to be a critical component of its oncogenic capability. For example, reactivation of the EBV lytic cycle is accompanied by decreased expression of MHC class I and class II molecules at the cell surface, protecting cells harboring replicating EBV from recognition and lysis by virus-specific cytotoxic T cells (CTLs) [64,65]. There is convincing evidence showing that an impaired MHC class I antigen processing pathway during the lytic cycle is an important mechanism to compromise the recognition of EBV-infected cells by CD8+ T cells [65,66,67]. A number of lytic genes are thought to contribute to these immunoregulatory effects.

#### 4.1.1. BNLF2a

It was shown in 2005 that the CD8+ T cell recognition of EBV-infected B cells undergoing the lytic cycle was dramatically reduced with progress through the lytic cycle, accompanied by a reduction in transporter associated with antigen processing (TAP) function and surface expression of HLA class I [64,67]. Screening of EBV gene products for their involvement in this process identified BNLF2a, which efficiently disrupted CTL-mediated cell lysis through HLA-A, -B, and -C alleles [66]. This study also showed that BNLF2a down-regulated levels of MHC class I at the cell surface and significantly blocked TAP function. TAP is a member of the family of ABC transporters that translocate peptides from the cytosol into endoplasmic reticulum (ER) for binding to newly synthesized MHC class I molecules and for subsequent presentation to CD8+ T-cells [68]. TAP is composed of two subunits, TAP1 and TAP2, that form a structure consisting of a C-terminal domain mediating ATP binding. The transport of peptides across the ER membrane by TAP depends on ATP hydrolysis [69]. BNLF2a directly interacted with the TAP complex and inhibited both its peptide- and ATP-binding functions, resulting in the failure of TAP to transport peptides into ER lumen [66]. Subsequent experiments showed that BNLF2a is an early lytic protein which co-localizes with TAP primarily in the ER [53]. More specifically, BNLF2a was characterized as a tail-anchored (TA) protein where a cellular protein called Asna1 was responsible for integration of BNLF2a into the ER membrane, and thereby required for BNLF2a-mediated HLA-I down-regulation [70]. The knockout of BNLF2a led to better CTL recognition of immediate early and early antigens, but not the late antigens, suggesting that BNLF2a expression is stage-specific and predominantly hampers the presentation of immediate early and early proteins [71].

Several research groups have independently showed that BNLF2a is expressed in the early phase of the lytic cycle in B cells, and that its expression is regulated by Zta [53,71,72,73]. Unexpectedly, Strong and colleagues demonstrated that *BNLF2a* is expressed in a substantial proportion of EBVaGCs and that this is independent from lytic gene expression [54]. This was the first report describing *BNLF2a* expression along with EBV latent genes and prompted the researchers to propose a new form of the type II latency program, latency IIc (EBNA1, LMP2 and BNLF2a). Subsequently, *BNLF2a* has also been shown to be expressed in primary NPC biopsies and patient-derived NPC xenografts [12,17,74]. Somatic aberrations in genes related to innate and adaptive immunity were identified through whole genome profiling of primary NPC [74]. Interestingly, a trend for mutual exclusivity between *BNLF2a* expression and these somatic alterations was observed, suggesting a host-viral cooperation for NPC cells to avoid immune detection. In accordance with these observations, single-cell transcriptomic analysis also identified a strong correlation between the expression of *BNLF2a/2b* and the host genes involved in immune responses [17]. By analyzing publicly available RNAseq datasets, 4 out of 1017 non-small cell lung cancer were found to be EBV-positive [75]. Notably, high expression of *BNLF2a* was detected in the sample with the highest EBV read number in the absence of other lytic gene expression. In addition to carcinomas, *BNLF2a* was also expressed in extra nodal natural killer T cell lymphoma (ENKTCL) and angio-immunoblastic T cell lymphoma (AITL) that exhibit latency IIc [6,13,16]. Taken together, these data strongly implicate BNLF2a in the oncogenic process aside from its contribution to the immune evasion of lytically-infected cells.

#### 4.1.2. BGLF5

A novel nuclease activity mapping to *BGLF5* gene was originally reported in a Burkitt lymphoma (BL) cell line, P3HR-1 [76], with a subsequent study identifying the BGLF5 gene product as an alkaline exonuclease [77]. Following the discovery that Kaposi sarcoma herpesvirus (KSHV) ORF37 encodes a host shutoff function [78,79,80], BGLF5 was also found to inhibit cellular protein synthesis [81]. Viral host shutoff (vhs) is an important mechanism exploited by viruses to curb cellular protein production thereby favoring the synthesis of viral proteins. Herpesviruses use this mechanism to down-regulate surface expression of MHC class I and II molecules, perturbing T cell recognition to achieve immune evasion [82]. Indeed, BGLF5 was capable of causing a widespread shutoff of cellular gene expression by enhanced mRNA degradation, including HLA class I and class II molecules [81]. Consequently, a marked impairment of HLA class I-restricted CD8+ T cell recognition was evident [83]. Although the shutoff function of BGLF5 was originally found to be genetically separable from its exonuclease activity [83], subsequent mutational analysis showed that its DNase and RNase activities share a catalytic site, and some mutations were more selectively affecting either DNA degradation or shutoff [84]. It was also shown that BGLF5 expression leads to nuclear relocalization of cytoplasmic poly(A) binding protein (PABPC), a protein involved in mRNA stabilization, thereby augmenting its shutoff phenotype [84]. Further detailed investigations revealed that BGLF5 is a component of virus-induced nodular structures (VINORCs) that contribute to the vhs-associated blockade of nuclear export of cellular mRNA while facilitating selective processing and export of viral mRNAs [85].

Cross-talk between the innate and adaptive immune system is required for the successful elimination of most pathogens. Upon the recognition of pathogen-associated molecular patterns, toll-like receptors (TLRs) activate intracellular signaling networks to either achieve direct antiviral activity or orchestrate the adaptive immune response [86]. Notably, BGLF5 was found to downregulate TLR9 levels through RNA degradation, a mechanism by which EBV could obstruct the host’s innate response [87]. Knockdown of BGLF5 in reactivated Akata BL cells restored the levels of several immunologically relevant molecules, such as TLR2, HLA class I/II molecules and CD1d [88]. 

In addition to its ability to facilitate immune evasion, BGLF5 also directly contributes to viral DNA replication and virion assembly [89]. It was found that knockout of BGLF5 resulted in a significant reduction of virus production by impairing virus nucleocapsid maturation, reducing primary egress and viral DNA synthesis. BGLF5 is expressed early in the viral replicative cycle [81,83]. Antibodies against BGLF5 were detected in sera from NPC patients [90] and BGLF5 protein expression, as well as nuclease activity, have been documented in both NPC biopsies and transplanted tumor lines [91]. In support of these observations, recent RNAseq analysis also revealed *BGLF5* expression in primary NPC tissues [12], but the precise levels and proportion of tumor cells expressing *BGLF5* remain to be determined. It is noteworthy that so far, there is no report describing BGLF5 expression in other EBV-associated malignancies. It remains to be determined whether the expression of BGLF5 is restricted to EBV-associated epithelial malignancies. 

#### 4.1.3. BILF1

BILF1 was initially identified as a putative G protein-coupled receptor (GPCR) based on its homology with the equine herpesvirus 2 E6 viral GPCR gene [92], but its functional role as a GPCR was not examined until 2005 [93,94]. These studies revealed that BILF1 is a constitutively active GPCR which signals through Gαi, classifying it as an “orphan” receptor. BILF1 contains seven transmembrane (TM) helices and displays conserved cysteine residues in the N-terminus and in the extracellular loops (ECLs). However, unlike most rhodopsin-like GPCRs (Class A GPCRs) which comprise a DRY (aspartic acid, arginine, tyrosine) motif in TM-III that is essential for receptor signaling, BILF1 has an alternative DRY motif, EKT (glutamic acid, lysine, threonine) [94]. Functioning as a ligand-independent GPCR, BILF1 is capable of modulating a number of intracellular pathways, including NF-kB and cAMP-response element-binding protein (CREB) pathways [93,94]. *BILF1* was originally identified as an early lytic gene [95], but its expression has also been detected in various primary tumor biopsies alongside the predominant latent mode of EBV infection [9,11,12,15,16,17,18,96,97].

The contribution of BILF1 to immune evasion was initially suggested by the observation that its expression resulted in the reduction of RNA-dependent protein kinase (PKR) activation [93]. This interferes with the cellular antiviral defense system in which PKR functions by shutting down host protein synthesis and inducing apoptosis of infected cells to halt virus spread [98]. Subsequently, BILF1 was found to down-regulate MHC class I molecules on the surface of host cells, making it the third EBV lytic gene identified to subvert the antigen presentation pathway [99]. BILF1 physically interacts with MHC-I molecules through its C-terminal tail that leads to accelerated internalization and lysosomal degradation, resulting in reduced levels of MHC-I at the cell surface and consequently abrogation of T-cell recognition [95,99]. The underlying mechanism for this effect is rather complex, but the conserved residues in both the EKT motif and ECL domains appear to play important roles [100,101]. Significantly, BILF1 can selectively reduce cell surface levels of HLA-A, -B, and -E alleles, while only marginally affecting HLA-C [95]. Through this refined modulation, BILF1 could interfere with HLA-A and –B presentation to CD8+ T-cells while enabling the infected cells to retain the inhibitory effect of HLA-C on NK cells, evading both adaptive and innate immune responses. In addition to the enhanced endocytosis of surface MHC-I molecules, BILF1 also diverts newly synthesized MHC-I/peptide complexes during exocytosis, resulting in a significant reduction of processed target peptides presented to CD8+ T-cells [100].

There is evidence to show that heterodimerization of two different GPCRs can alter the functional features of the individual partners, including signaling and trafficking [102]. Interestingly, BILF1 has been shown to form heterodimers with several human chemokine receptors [103], a strategy that EBV might use to hijack cellular communication for its own benefit. In particular, BILF1 hetero-oligomerizes with human CXCR4 to disrupt binding of CXCL12 to CXCR4 in a constitutive manner, resulting in the impairment of CXCL12-mediated CXCR4 signaling [104]. Whist the functional significance of this effect has yet to be explored, the authors postulated that the migration of BILF1-expressing plasma B cells to CXCL12 gradients could be suppressed, and this might provide an advantage for EBV by homing to sites that are most optimal for viral replication and dissemination. Nonetheless, it is noteworthy that CXCR4 has been shown to cause a reduction of MHC class I levels at the cell surface [105]. The involvement of CXCR4 in BILF1-mediated down-regulation of MHC-I warrants further investigation.

In the context of oncogenesis, another intriguing property of BILF1 is its function as an oncogene [106]. BILF1 was shown to induce foci in NIH3T3 cells in vitro, as well as tumor formation in vivo through EKT-dependent Gαi signaling. BILF1 also stimulated vascular endothelial growth factor (VEGF) secretion in a constitutively active manner [106]. The contribution of VEGF to tumor angiogenesis is well-defined, and its up-regulation has been reported in many tumors, including NPC and non-Hodgkin lymphoma [107,108]. In support of its tumor-promoting properties, BILF1 has been demonstrated to up-regulate intercellular adhesion molecule-1 (ICAM-1) [109]. The up-regulation of ICAM-1 has been described in various types of cancer [110] and can promote cancer metastasis [111]. Site-directed mutagenesis of the NF-kB binding sites on the *ICAM-1* promoter significantly diminished the ability of BILF1 to up-regulate ICAM-1, suggesting BILF1 exerts its function through a mechanism involving the NF-kB pathway [109]. Further experiments demonstrated that BILF1 decreased the cellular levels of IkBα (an inhibitory molecule of NF-kB), and this likely resulted in the translocation of NF-kB from cytoplasm to nucleus. Taken together with the detection of BILF1 in EBV-associated tumors, these results imply a significant role for this protein in the pathogenesis of these malignancies.

During EBV replication, CD8+ T cell responses to EBV immediate-early and some early antigens are more frequently observed than responses to late antigens. This hierarchy of immunodominance seems to correlate with the phase-specific efficiency of antigen presentation that is modulated by the viral immuno-evasins. Knockdown experiments in LCLs have shown that BNLF2a impairs antigen presentation with decreasing efficiency as the lytic cycle progresses while subversion by BILF1 increases with progression through lytic cycle [112]. In contrast, BGLF5 has a relatively minor effect on CD8+ T cell recognition of antigens expressed in any phase of lytic cycle [112].

#### 4.1.4. BCRF1

The *BCRF1* gene encodes viral interleukin-10 (vIL-10), a human homolog of interleukin-10 (hIL-10) that is commonly regarded as an immunosuppressive cytokine [113,114]. *BCRF1* was originally thought to be expressed only during the late phase of the lytic cycle, however, its expression has also been detected immediately after the infection of primary B cells [73,115]. A viral pre-initiation complex (vPIC) comprising several early lytic proteins (also known as “late gene regulators”) is required for the transcription of late genes [116]. Interestingly, *BCRF1* and another late gene, *BPLF1*, are transcribed independently of the vPIC [117], a mechanism different from that regulating late genes encoding structural proteins. Notably, the *BRLF1*-encoded transcription activator (Rta) selectively binds to eight late promoters, including *BCRF1*, suggesting the expression of *BCRF1* is likely regulated by *BRLF1* [117]. Significantly, an early in vitro study showed that vIL-10 was present in EBV-transformed cell lines and that *BCRF1* antisense oligonucleotides inhibited B cell transformation, pointing to a critical role for *BCRF1* in EBV tumorigenesis alongside the well-established latent genes [115]. However, a study published around the same time reported that vIL-10 was not essential for B cell transformation in vitro [118]. Nonetheless, the expression of *BCRF1* was reported in primary tumors of NK/T lymphoma and BL [31,119] and is now evidenced in various types of EBV-associated malignancies (Table 1). Notably, Badya and colleagues [16] reported that most AITL patients co-express *BNLF2a* and *BCRF1*, supporting a previous in vitro study showing that co-expression of these two genes facilitated immune evasion during the early phase of EBV lytic infection [73].

The immunosuppressive properties of vIL-10 were revealed by a number of studies in the 1990s. In a murine model, infection with a recombinant vaccinia virus expressing vIL-10 resulted in reduced NK and CTL responses, suggesting that BCRF1 may facilitate the establishment of latent infection in B lymphocytes [120]. These results were supported by subsequent experiments, showing that vIL-10 protected EBV-infected B cells from NK cell-mediated killing [73]. Similar to hIL-10, vIL-10 functions as a cytokine synthesis inhibitory factor that blocks interferon-γ (IFN-γ) synthesis by activated lymphoid cells [121]. Furthermore, vIL-10 was shown to interfere with the antigen presentation pathway by down-regulating TAP1 to hamper the transport of peptide antigens into the ER, which in turn reduced the surface levels of MHC-I levels, thereby contributing to the evasion of T cell recognition [122]. In addition, vIL-10 significantly inhibited antigen-specific CD4+ T-cell activation by impairing the antigen-presenting capacity of monocytes through down-regulation of MHC class II expression as well as adhesion/costimulatory molecules ICAM-1, CD80, and CD86 [123,124]. In accordance with these observations, vIL-10 has been shown to abrogate the ability of autologous T cells to inhibit EBV-induced B cell transformation [125]. This effect appeared to be mediated through an augmentation of the growth of infected cells and the suppression of IL-2 and IFN-γ production induced by activated T cells [125]. In addition, vIL-10 prevented the secretion of anti-viral cytokines and diminished CD4+ effector T cell functions [73]. Collectively, these results indicate that EBV appears to have captured a human cytokine gene and retained activities necessary to enhance cell transformation during initial infection and to suppress host immune responses triggered by subsequent viral reactivation.

### 4.2. Genomic Instability

Genomic instability is generally defined by an increased frequency of genetic changes including nucleotide-excision repair-associated instability, microsatellite instability (MSI) and chromosomal aberration-associated instability [126]. Genetic instability is a well-recognized hallmark of cancer and, in this regard, EBV-associated malignancies are no exception [127,128]. Genomic instability results from defects in a variety of processes, including increased rates of DNA damage, impairment of repair systems, and defects in chromosomal segregation. It has been shown that EBV lytic replication induces the genetic instability of epithelial and B cells [129,130], and that this could contribute to the development of tumors even in situations where EBV infection is subsequently lost [130]. The mechanisms underlying EBV-induced genomic instability remain to be fully elucidated, although a number of lytic genes have been shown to promote this phenomenon in different ways.

#### 4.2.1. BGLF5 and BALF3

Both BGLF5 and BALF3 gene products possess DNA cleavage activity and have been shown to promote genomic instability through similar mechanisms. It has been shown that the degree of genomic instability (measured by micronuclei formation) and chromosomal aberration in NPC cells are proportional to the frequencies of EBV lytic reactivation upon chemical treatment [129,131]. Examination of a panel of EBV early genes that might contribute to genomic instability in NPC cells identified BGLF5 as a potent inducer of DNA double-strand breaks, and the formation of micronuclei [129]. Subsequent experiments confirmed that BGLF5 alone was able to trigger genomic instability in epithelial cells by directly inducing DNA damage and indirectly repressing DNA repair, leading to increased chromosomal aberrations, MSI, and genetic mutations [132].

BALF3 is a terminase that cleaves newly synthesized viral DNA and translocates unit length viral genomes into procapsids during EBV lytic cycle [133]. It plays an important role in mature virion production by simultaneously involving in DNA synthesis and packaging. Using NPC cells as a model system, BALF3 was shown to induce DNA strand breaks, formation of micronuclei, and accumulation of chromosomal aberrations [134]. These effects of BALF3 were dependent upon its nuclease activity. Notably, BALF3 also contributed to a number of malignant phenotypes of NPC cells, such as enhanced cell migration, invasion, and spheroid formation in vitro as well as the promotion of tumor growth in vivo [134]. 

#### 4.2.2. BNRF1

BNRF1 is a virion tegument protein that facilitates the transfer of virus particles containing the nucleocapsid from the endosome to the nucleus [89]. A role for BNRF1 in inducing chromosomal instability was demonstrated when infection of primary B cells with a wild-type EBV resulted in centrosome amplification and aneuploidy compared to cells infected by a lytic-defective variant [130]. Further experiments revealed that BNRF1 was responsible for these effects and, specifically, BNRF1 induced centrosome over duplication during the S phase. However, the mechanisms that underlie BNRF1-mediated chromosomal instability was unclear, although sucrose gradient fractionation experiments found BNRF1 to be enriched in the centrosomal fraction and BNRF1 increased the production of the truncated form of PARP1 (a protein in centrosome) generated by caspase cleavage within the centrosome. The structural maintenance of chromosomes (SMC) 5/6 play major roles in chromosome maintenance and DNA damage repair [135]. Recently, BNRF1 was reported to target SMC5/6 for proteasomal degradation via Cullin 7 and calpain [136].

BNRF1 is originally thought to be expressed exclusively in late lytic cycle [137]. In a study that set out to examine the issue of immunodominance among EBV lytic proteins in CD8+ T cell memory, it was unexpectedly found that BNRF1-specific T cell clones also recognized latently-infected, growth transformed LCLs, implying that BNRF1 was also expressed in EBV-infected B cells and possibly virus-associated lymphoproliferative disease [137]. It is noteworthy that BNRF1 has been shown to counteract the cellular antiviral defense machinery to facilitate the establishment of viral latency [63,138,139]. BNRF1 was shown to bind to the histone H3.3 chaperone DAXX and disassemble the DAXX-ATRX complex, in which DAXX and ATRX are cellular proteins that repress viral gene expression during latency. The BNRF1-DAXX interaction was also responsible for localizing BNRF1 to promyelocytic leukemia (PML)-nuclear bodies that are involved in host antiviral resistance and transcriptional repression, as well as promoting the expression of selective viral latent cycle genes required for EBV-induced B-cell proliferation and immortalization [63,138,139]. In support of these observations, *BNRF1* expression has been consistently detected in various EBV-driven malignancies by RNAseq analysis (Table 1). Notably, BNRF1 appears to be an immunodominant target recognized by EBV-specific cytotoxic CD4+/CD8+ T cells and CD4+T helper (Th) cells [137,140,141]. Interestingly, the EBV subtypes that are strongly associated with the risk of NPC in China contain four amino acid substitutions (BALF2 V317M, BNRF1 G696R, V1222I, and RPMS1 D51E) [142]. In particular, BALF2 V317M and BNRF1 V1222I showed the strongest evidence of positive selection, implying that the virus might have evolved with these genetic alterations to avoid host immune surveillance and promote carcinogenesis.

### 4.3. Cell Survival

Apoptosis and autophagy are biological processes that contribute to cellular defense against intracellular pathogens. Many pathogens have evolved complex molecular strategies to escape these defense mechanisms by inhibiting apoptosis and/or promoting autophagy. EBV encodes two viral homologs of the cellular anti-apoptotic Bcl-2 oncoprotein (vBcl-2), BHRF1, and BALF1 [143,144]. Both *BHRF1* and *BALF1* are classified as early lytic genes that prevent apoptosis during early infection of primary B cells, and function redundantly in driving the transformation of B cells [145]. However, accumulating evidence points to a role for these proteins in latently infected cells.

#### 4.3.1. BHRF1

Although *BHRF1* is classified as a lytic gene, its transcripts have also been detected during the latent phase of EBV infection. Early studies described the expression of *BHRF1* mRNA in tissue samples of Hodgkin, NK/T cell lymphoma and NPC [146,147]. However, it was thought that *BHRF1* was most likely expressed by rare cells in the tumors that had entered lytic cycle. The first evidence showing BHRF1 expression during latency came from research on BL [148]. It was shown that the expression of BHRF1 early during infection of B cells is independent of *BZLF1* and *EBNA2* [148]. Significantly, BHRF1 was constitutively expressed as a latent protein in Wp-restricted BL cells that unusually exhibited strong resistance to apoptosis, suggesting it might contribute to EBV-driven lymphoma genesis. The expression of *BHRF1* was also subsequently demonstrated in biopsies of DLBCL, NPC, and EBVaGC [48,100,149,150].

The anti-apoptotic properties of BHRF1 were identified by a series of studies. BHRF1 was initially shown to function in a way similar to Bcl-2 by suppressing apoptotic cell death induced by DNA damaging agents [151,152]. Subsequent studies showed that BHRF1 protected human epithelial cells from apoptosis induced by a wide range of stimuli, such as cisplatin, tumor necrosis factor alpha, anti-Fas, activated monocytes, and serum deprivation [153,154,155]. It also delayed the terminal differentiation of epithelial cells through the prevention of apoptosis [153], implicating a role in enhancing the survival of EBV-infected epithelial cells leading to the development of malignancy. In the context of BL, BHRF1 expression conferred potent resistance to multiple chemotherapeutic agents and the underlying mechanism has been ascribed to its ability to interact and inhibit several cellular pro-apoptotic Bcl-2 proteins, Bim, Bid, Puma and Bak [156,157,158]. Further, expression of BHRF1 in mouse hematopoietic stem and progenitor cells accelerated MYC-induced lymphoma development in a model of BL [158], further supporting a role for BHRF1 in tumorigenicity and the survival of tumor cells.

The impact of BHRF1 on promoting cell survival through mitochondrial regulation has also been described. Mitochondria play a central role in cell homeostasis by regulating both cell death and cell survival [159]. Apoptosis can be triggered intrinsically through mitochondrial pathways that are tightly controlled by Bcl-2 family members [160]. On the other hand, autophagy is a “self-eating” physiological pathway that promotes cell survival [161]. Mitochondrial autophagy is termed “mitophagy” [162]. It was found that BHRF1 preferentially localized to mitochondria and interacted with Bim to confer mitochondrial stability under apoptosis-inducing conditions [163]. In NPC cell lines, BHRF1 was able to induce mitochondrial membrane permeabilization transition (MMPT), resulting in intensified reactive oxygen species (ROS) production and subsequent activation of mitophagy [150]. This molecular scenario prevented apoptosis and favored NPC tumorigenesis. In addition, mitochondria are essential in the activation of IFN pathways against viral infection as the first line of the defense response in innate immunity [164]. It has been shown that BHRF1 disturbed mitochondrial dynamics by inducing mitochondrial fission and hyperacetylation of the microtubules (MT) that regulate movement of mitochondria in the cytoplasm [165,166]. Consequently, these effects led to the stimulation of mitophagy, resulting in the inhibition of IFN production. These data are in agreement with studies describing the ability of EBV to subvert autophagy thereby enhancing viral replication and counteracting host anti-viral immune responses [167,168]. 

#### 4.3.2. BALF1

Compared to BHRF1, BALF1 has been less studied, and its anti-apoptotic function remains to be confirmed. BALF1 was initially found to inhibit α-FAS/IFN-γ-induced apoptosis by interacting with Bax and Bak [144]. However, another study showed that BALF1 lacked anti-apoptotic activity and impaired the anti-apoptotic activity of BHRF1 [169]. It was speculated that like cellular Bcl-2 that can inhibit or induce apoptosis in part by counteracting the activity of other Bcl-2 family members, EBV also encodes an antagonist of its own Bcl-2. However, unlike the cellular Bcl-2 antagonists, BALF1 lacked pro-apoptotic activity [169]. Despite these conflicting observations, subsequent experiments demonstrated that BALF1 reduced the serum-dependence of cells and significantly increased cell survival under serum-starved conditions by suppressing apoptosis, but not promoting cell cycle progression [170,171]. In addition to its effects on apoptosis, BALF1 was shown to enhance cell migration and invasion in vitro and to promote tumor growth and metastasis in vivo [171]. 

Interestingly, similar to BHRF1, Shao and colleagues have highlighted the involvement of BALF1 in autophagy [172]. In addition to its role as a self-eating process, autophagy also emerged as a microbial clearance mechanism by presenting pathogenic antigens to the immune system [173]. It was shown that BALF0/1 can modulate autophagy during the EBV lytic cycle, possibly to favor the formation of viral particles. The potential contribution of BALF1 to immunomodulation via autophagy manipulation deserves further investigation. 

Due to the unavailability of a specific anti-BALF1 antibody, the expression of BALF1 protein in EBV-infected cells has never been confirmed. Nonetheless, *BALF1* mRNA has previously been documented in BL cell lines and NPC biopsies [170], and readily found in tumor samples of EBVaGC and NPC by RNAseq (Table 1). Perhaps more importantly, anti-BALF0/1 antibodies were detected in plasma samples of NPC patients, providing indirect evidence for the existence of BALF1 in vivo [172]. 

## 5. Conclusions Remarks

**Figure 2:**
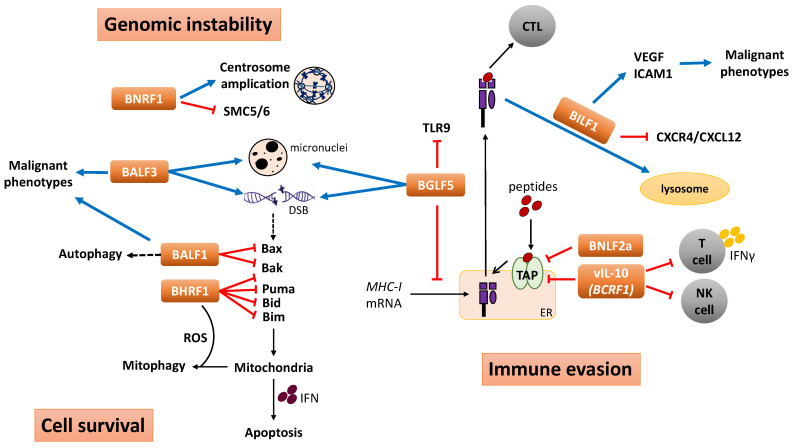
Contribution of EBV lytic gene products to the oncogenic process through immune evasion, induction of genomic instability and enhanced cell survival. BNLF2a, BGLF5, BILF1 and *BCRF1*-encoded vIL-10 impair the antigen presentation pathway to evade T cell recognition of EBV-infected cells. vIL-10 also reduces NK cell responses and suppresses T cell activity through inhibition of IFN-γ. Along with BALF3 and BNRF1, BGLF5 also induces genomic instability. BGLF5 and BALF3 induce DNA strand breaks and formation of micronuclei, while BNRF1 causes chromosomal aberrations by inducing centrosome amplification and degradation of SMC5/6 that is crucial in chromosome maintenance and DNA damage repair. In response to apoptosis, BHRF1 and BALF1 protect EBV-infected cells by inhibiting pro-apoptotic Bcl-2 proteins as well as subverting mitochondrial dynamics and regulating autophagy. In addition, BILF1, BALF1 and BALF3 can also promote the malignant phenotype of EBV-positive tumor cells. DSB, double-strand break.

Evidence is accumulating to suggest that EBV-encoded lytic genes could contribute to the oncogenic process through several coordinated mechanisms (Figure 2). Upon initiation of the lytic cycle, BNLF2a is produced to shut down TAP-mediated transport of antigenic peptides into the ER, thereby preventing peptide-loading of MHC-I molecules. BGLF5 acts synergistically to block the synthesis of new MHC-I molecules. *BCRF1*-encoded vIL-10 further compromises antigen presentation via the class I pathway. MHC-I complexes that have reached the cell surface are down-regulated by BILF1. Collectively, these events ensure effective interference with T cell recognition to avoid the elimination of viral infected cells. BGLF5 and BALF3 trigger genomic instability by inducing the formation of micronuclei and DNA strand breaks, while BNRF1 stimulates centrosome amplification resulting in chromosomal aberration. EBV-infected cells carrying genetic abnormalities escape host defense mechanisms by inhibiting apoptosis and modulating autophagy. BHRF1 and BALF1 promote the survival of EBV-infected cells by inhibiting several pro-apoptotic Bcl-2 proteins. Further, BHRF1 hijacks mitochondrial dynamics to stimulate mitophagy that favor cell survival. The effect of lytic gene expression in the carcinogenic process may not be restricted to the above genes/processes because the expression of several other lytic genes, such as *LF1-3*, is also frequently detected in EBV tumors (Table 1). However, limited information is available regarding the function of *LF1-3*, and further studies are warranted to explore the roles of these genes in EBV biology and oncogenesis.

The ability of EBV-encoded lytic genes to influence fundamental processes associated with carcinogenesis such as immune evasion, genomic instability, and cell survival challenges the conventional acceptance that only virus latent genes contribute to EBV-associated cancers. The degree to which EBV lytic genes contribute to the initiation of the oncogenic process versus the maintenance of the transformed phenotype remains to be determined. Nevertheless, variation of EBV lytic gene expression within the tumor microenvironment could impact local immune recognition and tumor cell survival. These effects could also be manifest in tumors that are predominantly EBV negative, but where a small proportion of carcinoma cells are susceptible to virus infection and sustain a lytic infection. This may explain the controversial association of EBV with breast cancer where abortive lytic infection has been reported, particularly in cases from Asian countries [174].

EBV strain variation in NPC also provides support for the role of the lytic cycle in carcinogenesis. Aside from the association of BALF2 variants with increased risk of NPC [175], genetic differences have also been observed in the Zp promoter and the *EBNA1* gene. Previous work has shown that a polymorphism in the promoter (Zp) driving expression of the *BZLF1* immediate early gene of EBV is common in virus isolates from NPC [176]. Subsequent works showed that this polymorphism, the so-called Zp-V3 variant, enhances EBV lytic reactivation to various stimuli [177]. Another study has demonstrated that EBNA1 derived from EBV strains present in NPC are less efficient at supporting replication and maintenance of the virus episome, leading to increased lytic virus replication [178]. These observations are consistent with work on a NPC-derived EBV strain (M81), which is more lytic and also displays enhanced tropism for epithelial cells [179]. Thus, it appears that the EBV strain present in NPC may be more replication-competent than previously considered, and that virus replication, aside from increasing the number of infected cells, may contribute to the carcinogenic process by virtue of the function of certain lytic genes. The presence of a more replicative form of EBV in NPC may explain why elevated antibody titres to late viral antigens have diagnostic and prognostic value in this tumor [180].

The evidence implicating EBV lytic genes in the pathogenesis of NPC and other virus-associated tumors is overwhelming. The precise contribution of EBV replication and lytic gene expression to the development and maintenance of the transformed phenotype requires more detailed studies using better reagents to assess expression within tumor biopsies and exploiting novel in vitro systems using recombinant forms of EBV. The most significant question is whether lytic gene expression within tumors influences the response to therapy, and whether targeting lytic genes has any therapeutic benefit. Future studies need to focus on how EBV gene expression relates to other factors within the tumor microenvironment (e.g infiltrating lymphocytes, cancer-associated fibroblasts) and how these impact the response to novel interventions such as immune checkpoint inhibition. This understanding offers exciting prospects for the development of individualized treatment strategies for patients with NPC and other EBV-associated tumors.

## Figures and Tables

**Table 1:** Lytic genes that are detected in primary tissues of EBV-associated cancers by RNAseq.

Gene Name	Kinetics	Lytic Function	Cancer Types	References
*BZLF1*	IE	Transactivator	GC, NPC, COAD, BL, DLBCL, PTCL	[6,7,8,9,10]
*BRLF1*	IE	Transactivator	GC, NPC, DLBCL	[7,8,10,11,12]
*BORF2*	E	Ribonucleotide reductase large subunit	BL, ENKTCL	[13,14]
*BSLF1*	E	Primase	GC, DLBCL	[7,15]
*BSLF2/BMLF1*	E	mRNA export factor ICP27 homolog	PTCL, DLBCL, AITL	[6,15,16]
*BALF1*	E	vBcl-2	GC, NPC	[8,11,15,17]
*BALF2*	E	Single-stranded DNA-binding protein	GC, NPC, COAD, BL, DLBCL, ENKTCL	[7,8,9,11,14,15,17,18]
*BALF3*	E	Terminase large subunit	GC, NPC, BL, DLBCL, AITL, ENKTCL	[11,12,14,15,16,17,18]
*BHLF1*	E	Involved in viral DNA synthesis	AITL, BL, DLBCL,	[14,15,16]
*BHRF1*	E	vBcl-2	GC, NPC, BL, DLBCL	[7,9,10,12,14,15]
*BMRF1*	E	DNA polymerase processivity factor	GC, COAD, BL, DLBCL, ENKTCL	[7,9,13,14,15]
*BALF5*	E	DNA polymerase catalytic subunit	GC, NPC, BL, DLBCL, ENKTCL	[7,8,11,12,14,15,18]
*BARF1*	E	Soluble decoy for CSF-1	GC, ENKTCL	[8,10,11,13]
*BBLF4*	E	Helicase	NPC, GC	[7,12]
*BILF1*	E	gp64, vGPCR	GC, NPC, BL, DLBCL, ENKTCL,	[9,11,12,15,17,18]
*BNLF2a*	E	Inhibtor of TAP	GC, NPC, BL, DLBCL, PTCL, AITL, ENKTCL	[6,9,11,13,15,16,17]
*BNLF2b*	E	Not reported	GC, NPC, DLBCL, PTCL, ENKTCL	[6,11,13,17,18]
*LF3*	E	Involves in viral DNA synthesis	GC, NPC, BL, AITL	[11,14,16,17]
*BMRF2*	E/L	Membrane proteins	BL, DLBCL, ENKTCL	[13,14,15]
*BCRF1*	L	vIL-10	GC, NPC, AITL, BL,	[7,9,10,12,16]
*BALF4*	L	Envelope glycoprotein B	GC, NPC, BL, DLBCL, ENKTCL	[7,8,9,11,12,14,15,17,18]
*BKRF2*	L	gL, gp25	BL, DLBCL	[14,15]
*BLLF1*	L	gp350/220	GC	[7,10]
*BNRF1*	L	Major tegument protein	GC, NPC, BL, AITL, ENKTCL	[8,11,12,13,14,16,17,18]
*BCLF1*	L	Major Capsid Protein	NPC, GC	[7,12]
*LF1*	Unknown	Not reported	GC, NPC, DLBCL	[8,11,12,15,17,19]
*LF2*	Unknown	Protein that binds Rta	GC, NPC, BL, DLBCL	[8,9,11,12,14,15,17]

GC gastric cancer, NPC nasopharyngeal carcinoma, COAD colon adenocarcinoma, BL Burkitt lymphoma, DLBCL diffuse large B cell lymphoma, AITL angio-immunoblastic T cell lymphoma, PTCL peripheral T cell lymphoma, ENKTCL extra nodal natural killer T cell lymphoma.

**Table 2:** Detection of proteins or antibodies against EBV lytic genes that are expressed in primary EBV-associated cancers by RNAseq.

Gene Name	Samples	Assays	References
*BZLF1*	Biopsy, FNA, cell line	IHC, ICC, WB	[31,35,36,37]
*BRLF1*	Plasma, cell line	ELISA, WB, IF	[37,38,39,40]
*BORF2*	Plasma	ELISA	[38]
*BSLF1*	Cell line	WB	[41]
*BSLF2/BMLF1*	Plasma, cell line	Protein array, WB	[38,42,43]
*BALF2*	Biopsy, plasma	IHC, ELISA	[38,44]
*BHLF1*	Cell line	IF	[45]
*BHRF1*	Biopsy, plasma	IHC, WB, protein array	[38,46,47]
*BMRF1*	Biopsy, serum, saliva, cell line	IHC, ELISA, WB	[37,48,49,50]
*BALF5*	Plasma, cell line	Protein array, WB	[38,51]
*BARF1*	Biopsy	WB	[52]
*BBLF4*	Cell line	WB	[41]
*BNLF2a*	Cell line	WB, IF	[53,54]
*LF3*	FNA, biopsy	ICC, IHC	[31,36]
*BMRF2*	Cell line	WB, IF	[55]
*BCRF1*	Allograft, serum	IHC, ELISA	[56,57]
*BALF4*	Plasma, serum	Protein array, ELISA	[38,58]
*BKRF2*	Cell line	IP	[59]
*BLLF1*	Serum, cell line	IF, WB	[60,61,62]
*BNRF1*	Cell line	WB	[63]
*LF2*	Plasma	ELISA	[38]

FNA fine needle aspiration, IHC immunohistochemistry, ICC immunocytochemistry, WB western blotting, ELISA enzyme-linked immunosorbent assay, IF immunofluorescence, IP immunoprecipitation.
